# Supplementary material for: Root Mean Square Minimum Distance as a Quality Metric for Stochastic Optical Localization Nanoscopy Images
Source: Sci Rep. 2018 Nov 21;8:17211. doi: 10.1038/s41598-018-35053-8 (PMC6249279; doi:10.1038/s41598-018-35053-8)
Supplement: Supplementary file 1 — Appendix [file 41598_2018_35053_MOESM1_ESM.docx]

Root Mean Square Minimum Distance as a Quality Metric for Stochastic Optical Localization Nanoscopy Images

**Yi Sun**

Electrical Engineering Department, Nanoscopy Laboratory, The City College of City University of New York, New York, NY 10031, USA.

E-mail: ysun@ccny.cuny.edu

Supplementary Appendix

Proof of Property 7

Consider $d_{i}>0$ for $i=1,\ldots,N$. For $1\leq M<N$, if $M^{-1}\sum_{i=1}^{M} d_{i}>{(N-M)}^{-1}\sum_{i=M+1}^{N} d_{i}$, then by simple algebra it is obtained that

$$\frac{1}{N-M}\sum_{i=M+1}^{N} d_{i}<\frac{1}{N}\sum_{i=1}^{N} d_{i}. (ALISTNUM “Equation” \backslash L 1)$$

Condition (i) means that for any $s\in S$, if $x\in X$ minimizes $\left\| x-s \right\|^{2}$, then $x\notin X^{'}$. In other words, no $x\in X^{'}$ is involved in the first sum term in $D(X,S)$ of Eq. (1). Condition (ii) states that

$$\frac{1}{\left| X^{'} \right|}\sum_{x\in X^{'}} \min_{s\in S} \left\| s-x \right\|^{2}>\frac{1}{\left| {X-X}^{'} \right|+\left| S \right|}\left( \sum_{s\in S} \min_{x\in X-X^{'}} \left\| x-s \right\|^{2}+\sum_{x\in{X-X}^{'}} \min_{s\in S} \left\| s-x \right\|^{2} \right). (ALISTNUM “Equation” \backslash L 1)$$

It follows from Eq. (A1) that

$$\frac{1}{\left| {X-X}^{'} \right|+\left| S \right|}\left( \sum_{s\in S} \min_{x\in X-X^{'}} \left\| x-s \right\|^{2}+\sum_{x\in{X-X}^{'}} \min_{s\in S} \left\| s-x \right\|^{2} \right)$$

$$<\frac{1}{\left| X \right|+\left| S \right|}\left( \sum_{s\in S} \min_{x\in X} \left\| x-s \right\|^{2}+\sum_{x\in X} \min_{s\in S} \left\| s-x \right\|^{2} \right), (ALISTNUM “Equation” \backslash L 1)$$

that is, $D\left( X-X^{'},S \right)<D(X,S)$. (Q.E.D.)

Proof of property 8

Since

$$\frac{1}{\left| S^{*} \right|}\sum_{i=1}^{\left| S^{*} \right|} \left\| x_{i}^{*}-s_{i}^{*} \right\|^{2}<\frac{1}{\left| X \right|+\left| S \right|-2\left| S^{*} \right|}$$

$$\times\left( \sum_{s\in S} \min_{x\in X} \left\| x-s \right\|^{2}+\sum_{x\in X} \min_{s\in S} \left\| s-x \right\|^{2}-2\sum_{i=1}^{\left| S^{*} \right|} \left\| x_{i}^{*}-s_{i}^{*} \right\|^{2} \right), (ALISTNUM “Equation” \backslash L 1)$$

it follows from Eq. (A1) that $D\left( X^{*},S^{*} \right)<D(X,S)$. (Q.E.D.)
